# SPS and DPS: Two New Grid-Based Source Location Privacy Protection Schemes in Wireless Sensor Networks

**DOI:** 10.3390/s19092074

**Published:** 2019-05-04

**Authors:** Qiuhua Wang, Jiacheng Zhan, Xiaoqin Ouyang, Yizhi Ren

**Affiliations:** 1 School of Cyberspace, Hangzhou Dianzi University, Hangzhou 310018, China; renyz@hdu.edu.cn; 2 School of Communication Engineering, Hangzhou Dianzi University, Hangzhou 310018, China; zhanjiacheng@hdu.edu.cn (J.Z.); xiaoqin_ouyang@163.com (X.O.)

**Keywords:** wireless sensor network, privacy preservation, phantom node, random routing

## Abstract

Wireless Sensor Networks (WSNs) have been widely deployed to monitor valuable objects. In these applications, the sensor node senses the existence of objects and transmitting data packets to the sink node (SN) in a multi hop fashion. The SN is a powerful node with high performance and is used to collect all the information sensed by the sensor nodes. Due to the open nature of the wireless medium, it is easy for an adversary to trace back along the routing path of the packets and get the location of the source node. Once adversaries have got the source node location, they can capture the monitored targets. Thus, it is important to protect the source node location privacy in WSNs. Many methods have been proposed to deal with this source location privacy protection problem, and most of them provide routing path diversity by using phantom node (PN) which is a fake source node used to entice the adversaries away from the actual source node. But in the existing schemes, the PN is determined by the source node via flooding, which not only consumes a lot of communication overhead, but also shortens the safety period of the source node. In view of the above problems, we propose two new grid-based source location privacy protection schemes in WSNs called grid-based single phantom node source location privacy protection scheme (SPS) and grid-based dual phantom node source location privacy protection scheme (DPS) in this paper. Different from the idea of determining the phantom node by the source node in the existing schemes, we propose to use powerful sink node to help the source node to determine the phantom node candidate set (PNCS), from which the source node randomly selects a phantom node acting as a fake source node. We evaluate our schemes through theoretical analysis and experiments. Experimental results show that compared with other schemes, our proposed schemes are more efficient and achieves higher security, as well as keeping lower total energy consumption. Our proposed schemes can protect the location privacy of the source node even in resource-constrained wireless network environments.

## 1. Introduction

As an important part of the Internet of Things (IoT), Wireless Sensor Networks (WSNs) are widely used in civilian and military applications. A WSN is composed of two kinds of nodes, namely, the sensor node and the sink node (SN). The sensor node acts as an information source, sensing the existence of objects and transmitting data packets to the SN by communicating with adjacent nodes wirelessly. The sink node is a powerful node with high performance, that is, it has unlimited computing capacity, storage capacity and energy resources. The SN is used to collect all the information sensed by the sensor nodes [1].

In the monitoring task-driven WSN, sensor nodes sense the existence of monitored objects in their sensing region, the node closest to the monitored target is called the source node (SoN). Anytime, any sensor node may become a SoN. Once the target is detected, the SoN will generate the encrypted data packets and transmit them to the SN in a multi hop fashion [2,3]. However, due to the open nature of the wireless transmission medium, the packet sender can easily be located by the adversary. Therefore, although the enemy cannot obtain the content of the encrypted data packets, he can reversely go to the actual source nodes via hop-by-hop tracking along the routing path of the packets [4]. If the adversary gets the location of the actual source node, he can capture the protected objects, which may lead to unpredictable consequences. For example, a very important application of WSN is monitoring valuable objects or personnel. When a large number of sensor nodes are deployed in the field to monitor precious animals (such as pandas, South China tigers, and golden monkeys), the locations of the animals should not be learned by poaching. Similarly, on the battlefield, the location of the soldier should not be tracked by the enemy. In the process of monitoring these objects or personnel, it is necessary to protect the safety of the target while obtaining valid information. Therefore, the physical location privacy protection of the source node in WSN becomes a critical problem worthy to be studied.

**Figure 1:**
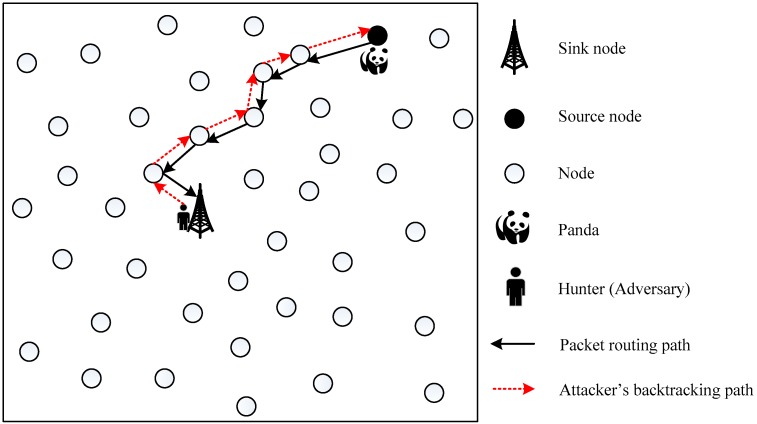
The Panda-Hunter model without privacy protection.

Source node location privacy protection is the process of keeping the location of a source node hidden from adversaries in a target monitoring network [5]. Celal Ozturk et al. first considered the source node location privacy problem of WSN in Reference [6], using Panda-Hunter game model, and based on this model they proposed to use a fake source called phantom node (PN) to entice the adversaries away from the actual source node [7]. In their Panda-Hunter model, shown in Figure 1, the monitored target is Panda, which is high-value and needs protection. In 2003, a single piece of panda fur was sold in Chongqing, China for $66,500 [8]. In Figure 1, a large number of sensor nodes are deployed in the field by the Save-The-Panda Organization to monitor the habitats of the panda [9]. Once the panda pops up, the sensor node closest to the panda becomes the SoN. The SoN generates the data packets and transmits them to the SN periodically via multi-hop routing techniques. The hunter (also featured as the adversary or attacker) starts at the sink node. He waits until he hears a packet. Once he hears a data packet, he can determine the packet sender via wireless locating technology [10] and quickly move to its location. By this way, the attacker can backtrack the routing path hop-by-hop to the SoN where the Panda is and capture the panda. In Figure 1, no source node location privacy protection technology is adopted, so the panda can be easily tracked and captured by the hunter. Therefore, the source location privacy protection technology must be used to prevent the hunter from locating the SoN, while ensuring that the data packets can be transmitted to the sink node.

Existing source node location privacy protection schemes protect source location privacy by increasing path length or path complexity, such as cyclic entrapment [11], dummy data sources [6,7,12,13,14,15,16], phantom routing [6,7,16,17,18,19,20,21,22], etc. These technologies mainly improve security performance by sacrificing network performance (such as increased communication overhead and decreased network stability). However, the communication overhead is the largest energy consumption and is far greater than the computation overhead [23]. For example, the energy consumed to send 1-bit data by a sensor node can be used to perform 3000 calculation instructions [24]. Therefore, how to save energy in the process of network operation to maximize the network life cycle is critical in the design of security strategy. Worse, the sensor node is powered by a battery and its computational capability, storage capacity and energy resources are limited, so the existing schemes are not applicable to such resource-constrained application scenarios. How to save energy in the process of protecting the source location privacy is critical when designing the security strategy.

In addition, in order to make the real source node difficult to be traced by the attacker, the PN should be as far as possible from the real source node [18], and the PN should be replaced periodically for the sake of security. However, in the existing schemes, the selected PNs are mainly concentrated near the SoN and remain unchanged, and the location privacy of the source node cannot be well protected.

### 1.1. Related Works and Issues

Since the exposure of the source node location in WSN inevitably threatens the security of the monitored target, the source node location privacy protection becomes an urgent issue to be solved. However, since the computational capability, storage capacity and energy resources of sensor nodes are limited, the balance between security and network performance becomes an inevitable requirement.

The existing researches on source node location privacy protection are mainly based on cyclic entrapment [11], dummy data sources [6,7,12,13,14,15,16] and phantom routing [6,7,16,17,18,19,20,21,22]. Ouyang et al. [11] introduced the cyclic entrapment concept as a special case of dummy data source routing. In cyclic entrapment, multiple nodes act as dummy data sources, and interconnect to form a loop. The main aim of cyclic entrapment is to confuse adversary with these loops during a hop-by-hop-trace attack, thereby preventing the attacker from going back to the real source node. However, such a strategy needs to activate one or more loops to restrict the attacker, and the nodes in the loop which act as the dummy data source need to generate dummy data periodically, which causes a large amount of abnormal communication overhead, results in energy hole [19] and damages the network performance seriously. In addition, when the Panda-Hunter model was first proposed in Reference [6], it has been assumed that the hunter has the ability to cache the location information, and he can record the position of the nearest *N* nodes, so as to avoid falling into the loop, which invalidates the protection capability of the scheme. Although dummy data source routing can protect source location privacy to a certain extent, it needs to generate a lot of dummy data periodically, which not only causes a lot of waste of energy consumption, but also increases packet collision probability and reduces packet transmission efficiency. Moreover, the true path of packet transmission is included in all paths, and the source node location privacy protection capability is probabilistic.

Ozturk et al. first proposed the phantom routing scheme (PRS) in Reference [6] to protect the location privacy of source nodes using the Panda-Hunter model. They use the phantom node to entice the adversaries away from the actual source node. If the attacker cannot track the PN, it is impossible for him to trace the location of the true source node [2]. PRS involves two phases: The random walk phase and the subsequent flooding phase. In the random walk phase, the packet from the real source is randomly transmitted *h* hops to determine a PN. In the flooding phase, the PN transmits the data packets to the SN by the way of flooding. The main aim of this scheme is to ensure that the attacker can only trace back to the PN and cannot trace back to the real source node, thus ensuring the privacy of the source node location. However, the shortest path in the flooding phase is included in the flooding paths, and the first packet received by the attacker is the shortest path packet. The attacker can capture the SoN by tracing back along the shortest path. Kamat et al. proposed a phantom single-path routing scheme (PSRS) in Reference [7], which is similar to the PRS scheme. In this scheme, the first phase is a directed random walk, and in the second phase, a single path routing algorithm is used to transmit data packets to the SN. This paper also provides a technique for classifying neighbors of nodes. In Reference [18], J. Chen et al. pointed out that the PNs in the existing source location privacy protection schemes are concentrated near the SoN, and once the attacker traces back to the PN, he can further trace to the SoN easily. In response to this problem, they proposed an enhanced source location privacy preservation protocol using source-based restricted flooding (EPUSBRF). Compared with the PSRS scheme, the EPUSBRF significantly improves the security of the source location privacy, and avoids the generation of the invalidate paths without increasing the energy consumption.

In recent years, the improvements for the source node location privacy protection of WSNs are mainly based on the idea of phantom routing. In Reference [19], Yi et al. proposed a trace time-constrained routing algorithm for preserving source-location in WSNs. Its main idea is that the routing path from the SoN to the SN is generated and distributed dynamically and randomly, and the lasting time of a routing path is shorter than the time for the adversary to trace to the PN. Hence, it is more difficult for the adversary to trace to the SoN, thus increasing the protection strength. In Reference [16], Ma et al. proposed a source location privacy preservation Routing Protocol Based on Multi-Path (RPBMP), which performs random routing based on multi-path selection and multiple PNs jump. This scheme greatly increases the number of routing path between the SoN and the SN, extends the safety period. The notion of the safety period is proposed in Reference [6] as a performance indicator of source location privacy protection in WSNs, and has been widely used in subsequent researches. In Reference [6], the safety period is measured by the number of packets the SoN has sent before it is caught by the attacker. To solve the problem of failure path in phantom routing protocols, in Reference [20], Chen et al. proposed an improved routing algorithm for WSN source node location privacy protection based on the minimum path routing. The improved routing algorithm has higher safety period and does better in protecting source location privacy in WSNs. In Reference [21], Kong et al. proposed a virtual ring-based routing protocol of source-location privacy protection, which avoids the generation of failure path and extends routing path to the annular region where SoN resided with the random virtual ring. This scheme greatly increases the diversity and randomness of the routing paths, and makes it harder for an attacker to deduce the SoN according to the routing path. In Reference [22], Wang et. al. proposed a location privacy protection strategy called PRABNS (Phantom Routing Based on Area and Brother Neighbor Selecting). The PNs selected by this strategy maintain a certain angle and distance, and the adjacent data packets have a certain angle space through the selection of partial region. This strategy increases the diversity for the path of the SoN to SN by selecting sibling nodes. However, the PNs determined by this strategy are still evenly distributed around the SoN, and the shielding nodes cannot guarantee the robustness of the network and the packet loss rate increases.

### 1.2. Our Motivations and Contributions

In this paper, we focus on the source node location privacy protection in WSNs, and our aim is to deal with the aforementioned problems, advance the existing researches and improve the security performance of source node. Different from the existing schemes which determine the PN by the SoN, in this paper, we propose to use powerful sink node to help the source node to determine the phantom node candidate set (PNCS) and propose two new grid-based source location privacy protection schemes in WSNs: The grid-based single phantom node source location privacy protection scheme (SPS) and the grid-based dual phantom node source location privacy protection scheme (DPS). In our proposed schemes, the PNCS is firstly determined by the SN, and then the SoN randomly selects a PN from the PNCS with equal probability. After the source node transmits the data packets to the selected PN via multi-hop routing, the PN sends the data packets to the SN via the single path routing. As the energy of SN is unlimited, the method that the SN helps the SoN to select the PNCS, not only avoids a large amount of energy consumed by the ordinary sensor nodes when determining the PN, but also distributes the PNs randomly throughout the network, thus greatly increases the security of the source node and provides better privacy preservation for them.

According to whether the PN is replaced, the above two schemes are further divided into two cases respectively: Not replacing the PN and replacing the PN. In the case of not replacing the PN, once the SoN chooses a PN from the PNCS, it will not be replaced. While in the case of replacing the PN, after a PN is used for a while, the SoN can replace the PN with another. When the PNCS is empty, that is, all the PNs selected by the SN are used up, the SoN sends a new request packet to the SN, requesting the SN to determine a new PNCS for it, which ensures that enough PNs can be used by the SoN during the panda’s stay. Moreover, since the SN knows about the basic location information of each node in the entire network, the PNs selected in our schemes are not concentrated near the SoN, but distribute anywhere in the network, and therefore have stronger positional randomness compared with other existing schemes. At the same time, as our schemes select more PNs to ensure the diversification of routing paths, the security performance of privacy protection is further improved. Our proposed new method greatly increases the security of the source node and provides better privacy preservation for them.

In this paper, the SPS scheme in the case of replacing the PN is called the RSPS scheme, the SPS scheme in the case of not replacing the PN is called the NRSPS scheme, the DPS scheme in the case of replacing the PN is called the RDPS scheme, and the DPS scheme in the case of not replacing the PN is called the NRDPS scheme.

In the performance simulation of Section 5, we explore the impact of replacing PNs on security performance, and the impact of the number of PNs on security performance and total communication overhead. Simulation results verify the effectiveness of our proposed schemes. For example, compared with the shortest path algorithm, RPBMP [16] and EPUSBRF [18], the average safety period of our proposed NRSPS scheme, is increased by 6.08 times, 2.78 times and 3.57 times respectively; compared with RPBMP and EPUSBRF, the total communication overhead of our proposed NRSPS scheme is reduced by 88% and 95%, respectively.

We summarize our main contributions as follows:

(1) We proposed two new grid-based source location privacy protection schemes. In our proposed schemes, the sink node with high-computing power and high-energy is used to determine the phantom node candidate set, which avoids the disadvantage of excessive communication overhead caused by flooding to determine the phantom node in previous traditional schemes;

(2) The location of the phantom nodes is determined by our proposed schemes that are distributed throughout the network; which, in turn, improves the diversity of the phantom nodes in terms of location and the randomness of routing. In our proposed schemes, the routing path appears randomly across the entire network, which prevents the attackers from backtracking to the location of the source node via the routing path. Our proposed schemes avoid the defects that the phantom node locations in the traditional schemes are concentrated near the real source node and that the location privacy of the real source node cannot be well protected;

(3) Our proposed schemes do not require the node to be equipped with a positioning module, such as GPS, which reduces the node cost and energy consumption, hence the application scenario of our proposed schemes are universal, and the source location privacy can be effectively and stably protected even in resource-constrained wireless network environments;

(4) We conduct theoretical analysis and extensive experimental simulations to evaluate the performance of our proposed schemes. The simulation results further verify the effectiveness of our proposed schemes.

### 1.3. Organization of the Paper

The rest of this paper is organized as follows. The system model, including the network model, attack model, and security model used in our proposed schemes, is introduced in Section 2. Section 3 provides a detailed description of our proposed SPS scheme, and Section 4 introduces our proposed DPS scheme. Section 5 presents the results and performance analysis. Finally, we conclude the paper in Section 6.

## 2. System Model

### 2.1. Network Model

Since the Panda-Hunter model was put forward in Reference [6] to study the source location privacy problem of WSNs, it has been widely used by researchers [6,7,11,12,13,14,15,16,17,18,19,20,25]. In this paper, similar to the existing researches, we also use the Panda-Hunter model as our network model, which is a deterministic deployment. For the sake of understanding, we make the following assumptions about the network model:

(1) There is only one sink node in the network, whose location is static and fixed in the network center. And the resources of the sink node, such as computing power, storage capacity and energy are not limited [26].

(2) The network is evenly divided into small grids. The sensor nodes in each grid are all fully connected. The whole network is fully connected through multi-hop communications [4].

(3) The sensor nodes in the network are deployed prior to the initialization phase. After being deployed, each node has the knowledge of its own basic information, such as node ID number, grid number, etc. The sink node knows about the basic information of each node in the entire network.

(4) When the panda appears, the node closest to the panda becomes the source node, that is, there is only one source node in the whole network. The source node will generate and send encrypted packets to the sink node through a multi-hop routing. The panda will stay for a period of time before leaving, and our schemes are applied during the panda’s stay period.

(5) To facilitate latter theoretical analysis and simulation, we assume that all sensor nodes in the network are the same type and any two nodes can communicate via multi-hop fashion.

(6) In order to ensure the universality of the scheme, for example, the scheme can be applied even in the resource-constrained wireless networks, the node does not have the positioning and mobility capabilities. Therefore, it cannot obtain its own precise coordinates and cannot directly calculate the actual distance between two nodes. Therefore, the hop count is used as the index to measure the distance. The distance between two nodes within the communication radius of each other is called one hop.

### 2.2. Attack Model

Due to the rarity of panda, the attacker is driven by interest and tries to use advanced equipment to capture the panda. During the panda’s stay period, the source node will continually send data packets, and the hunter may use this to his advantage to track and hunt the panda. Similar to most other pieces in the literature on source node location privacy protection [6,7,11,12,13,14,15,16,17,18,19,20,21,22], we mainly consider the local passive attackers with the ability to eavesdrop on local traffic of a WSN.

We make the following assumptions about the attack model:

(1) The attacker is equipped with wireless signal monitoring equipment, such as antenna and spectrum analyzers, and has sufficient computational capacity, storage capacity and energy resources. However, the attacker can only eavesdrop the network traffic in a local region; he cannot monitor the entire network. In fact, if the attacker can monitor the entire network, he can monitor the Panda directly without relying on the WSN [4]. Also, he cannot decrypt the packet and tamper with the packet content;

(2) The attacker just wants to get the location of the source node, in order to ensure his own concealment, the attacker only passively listens to the packets and hops back and forth. The attacker does not initiate an active attack on the network, that is, he does not interfere with the normal functioning of the network, and otherwise intrusion detection measures might detect the attacker’s presence;

(3) The initial position of the attacker is at the sink node. He waits until he hears a packet. Once the attacker hears a data packet, he can determine the packet sender via wireless locating technology [10] and quickly move to its location. The monitoring radius of the attacker is the communication radius of sensor nodes. Although the attacker has strong mobility, he can sense only one hop transmission, and he moves only when he monitored a data packet, that is, the attack tracks a packet only via hop-by-hop;

(4) We emphasize that the attacker cannot learn the origin of a packet by merely observing a relayed version of it. If the attacker does not overhead the data packet within a certain period of time, he will roll back hop-by-hop along the tracking path until he returns to the sink node;

(5) The monitored object can be captured when the attacker appears in the visible area of the source node.

### 2.3. Security Model

We make the following assumptions about the security of the network:

(1) The network has basic security measures, such as encrypting the data packet. The attacker cannot decrypt the packet, and can only capture the panda in the visible area of the source node via hop-by-hop backtracking. We will not discuss specific encryption and decryption algorithms and key management mechanisms, since they are beyond the scope of this paper.

(2) We assume that the source node includes its ID in the encrypted packets, but only the sink node can identify the source location from its ID. Even if the hunter can break the encryption in a reasonably short time, he cannot tell the source node’s location [4,8].

(3) The sink node is absolutely safe, and the attacker cannot break the sink node.

## 3. SPS: Grid-Based Single Phantom Node Source Location Privacy Protection Scheme

In this section, we first introduce the proposed grid-based single phantom node source location privacy protection scheme (SPS) in WSN. In our proposed schemes, the PNCS is firstly determined by the sink node, and then the source node randomly selects a PN from the PNCS. After the SoN transmits the data packets to the selected PN via multi-hop routing, the PN sends the data packets to the sink node via the single path routing. The method that the sink node helps the SoN to select the PNCS, not only avoids a large amount of energy consumed by the ordinary sensor nodes when determining the PN, but also distributes the PNs randomly throughout the network. Our proposed new method greatly increases the security of the SoN and provides better privacy preservation for them. In our latter description, the sensor node is simply referred to as nodes.

Specifically, our proposed scheme is divided into three phases: The initialization phase, the phantom node determination phase and the routing phase. For ease of understanding, the notations used herein are listed in Table 1.

### 3.1. The Initialization Phase

**Figure 2:**
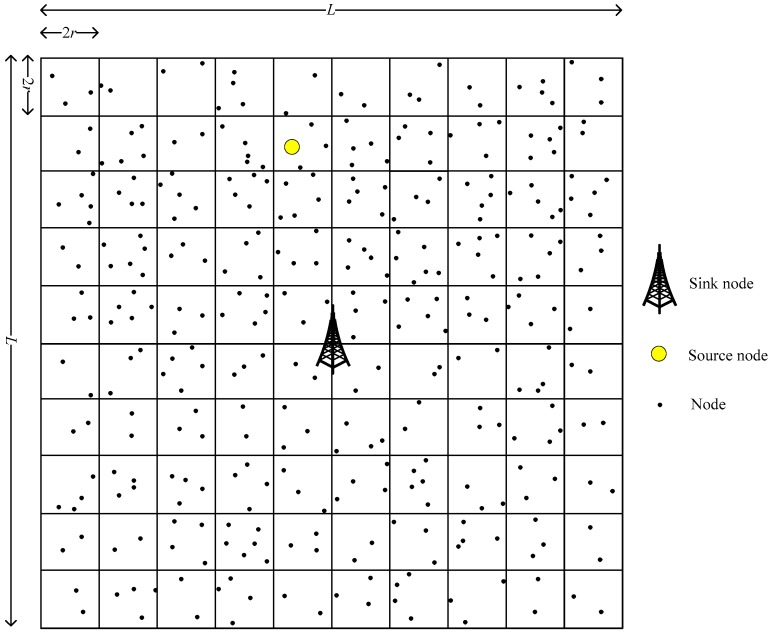
Network grid.

The specific process of the network initialization phase is as follows:

(1) As shown in Figure 2, the SN evenly divides the network into L*L grids with the unit length of 2*r* (*L* is an even number). The SN is fixed at the center of the network, and its grid number is represented as $G_{\frac{L}{2}\times \frac{L}{2}}$. The grid number of other grids is represented by the center point coordinates of the grid;

(2) The SN broadcasts a message Msg_b with the same power as the sensor nodes.

Sink node’s broadcast:

(1) $${\mathrm{Msg}\text{\_}b=\mathrm{ID}}_{\mathrm{sink}}{||\mathrm{Hop}}_{\mathrm{sink},\mathrm{sink}}||G_{\frac{L}{2}\times \frac{L}{2}}.$$

Message Msg_b includes three parameters. The first one is the ID of the sending node, here is ${\mathrm{ID}}_{\mathrm{sink}}$. The second one is the hop count of the sending node from the SN, here is ${\mathrm{Hop}}_{\mathrm{sink},\mathrm{sink}}=0$. The third one is the grid number of the sending node, here is $G_{\frac{L}{2}\times \frac{L}{2}}$;

(3) Suppose that node *u* is in grid $G_{i\times j}$. When node *u* receives the broadcast message Msg_b, it updates the minimum hop count of itself from the SN ${\mathrm{Hop}}_{u,\mathrm{sink}}$, and then continues to broadcast the message Msg_b to all of its neighbor nodes.

Node *u*’s broadcast:

(2) $${\mathrm{Msg}\text{\_}b=\mathrm{ID}}_{u}{||\mathrm{Hop}}_{u,\mathrm{sink}}{||G}_{i\times j};$$

(4) Suppose that the neighbor node *v* of node *u* is in the grid $G_{m\times n}$. When node *v* receives the broadcast message sent by node *u*, it adds the node ID of node *u*, the hop count of node *u* from the SN ${\mathrm{Hop}}_{u,\mathrm{sink}}$ and the grid number $G_{i\times j}$ of node *u* to its neighbor table, as shown in Table 2. Specifically, node *v* updates the minimum hop count of itself from the SN ${\mathrm{Hop}}_{v,\;\mathrm{sink}}$ and then continues to broadcast messages Msg_b to all of its neighbors.

Node *v*’s broadcast:

(3) $${\mathrm{Msg}\text{\_}b=\mathrm{ID}}_{v}{||\mathrm{Hop}}_{v,\mathrm{sink}}{||G}_{m\times n}.$$

Similarly, node *u* can also receive the broadcast message of node *v*, and will add the node ID of node *v*, the hop count of node *v* from the SN ${\mathrm{Hop}}_{v,\mathrm{sink}}$, and the grid number of node *v* to its own neighbor table;

(5) When all nodes receive the broadcast packet, the initialization phase ends. At this time, each node establishes its own neighbor table according to the received broadcast message. Then each node classifies its neighbor nodes into three categories. Taking node *u* as an example, it classifies its neighbor node *v* according to ${\mathrm{Hop}}_{u,\mathrm{sink}}$ and ${\mathrm{Hop}}_{v,\mathrm{sink}}$:

- Near-hop neighbor node: ${\mathrm{Hop}}_{v,\mathrm{sink}}<{\mathrm{Hop}}_{u,\mathrm{sink}}$;
- Same-hop neighbor node: ${\mathrm{Hop}}_{v,\mathrm{sink}}{=\mathrm{Hop}}_{u,\mathrm{sink}}$;
- Far-hop neighbor node: ${\mathrm{Hop}}_{v,\mathrm{sink}}>{\mathrm{Hop}}_{u,\mathrm{sink}}$.

### 3.2. The Phantom Node Determination Phase

**Figure 3:**
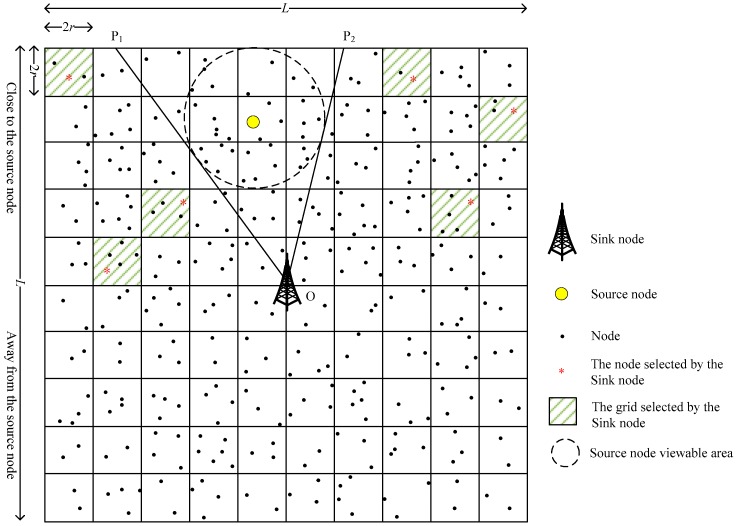
The sink node helps select phantom node candidate set.

(1) When the monitored target appears in the network, the node closest to the target becomes the source node. The SoN sends a request packet Msg_request to the SN, requesting the SN to help it determine the PNCS. The transmission mode of the request packet is: The SoN randomly selects a near-hop neighbor node from its neighbor table as the next hop node. The next hop node also randomly selects a near-hop neighbor node as its own next hop node. This process continues until the request packet reaches the SN;

(2) As shown in Figure 3, after the SN receives the request packet, it randomly selects *M* grids from the grids near the SoN while not in the visible area of the SoN (the grids outside the $\hat{P_{1}{\mathrm{OP}}_{2}}$ area). Then, the SN randomly selects one node in each of the selected *M* grids to form a PNCS;

(3) The SN sends the PNCS and the grid number of each node in the PNCS to the SoN;

(4) The SoN randomly selects one node from the PNCS as the actually used PN with equal probability. All the nodes within the PNCS are chosen with equal probability.

### 3.3. The Routing Phase

The SoN periodically sends encrypted data packets containing the panda information to the SN through the PN via multi-hop routing. The data packets need to go through two transmission steps: The SoN sends the data packets to the PN, and the PN sends the data packets to the SN.

#### 3.3.1. Step 1: The Source Node Sends Data Packets to The Phantom Node

In the process of sending data packets to the PN, the SoN only knows the ID of the PN and the grid number it is in. How to send data packets to the PN via multi-hop routing when no positioning capability is available is a question worth exploring. In this paper, we design the following strategies to solve this problem. Firstly, determine which grid will be passed through during the transmission process, and then find the node in the grid to forward the data packets by searching the neighbor table.

**Figure 4:**
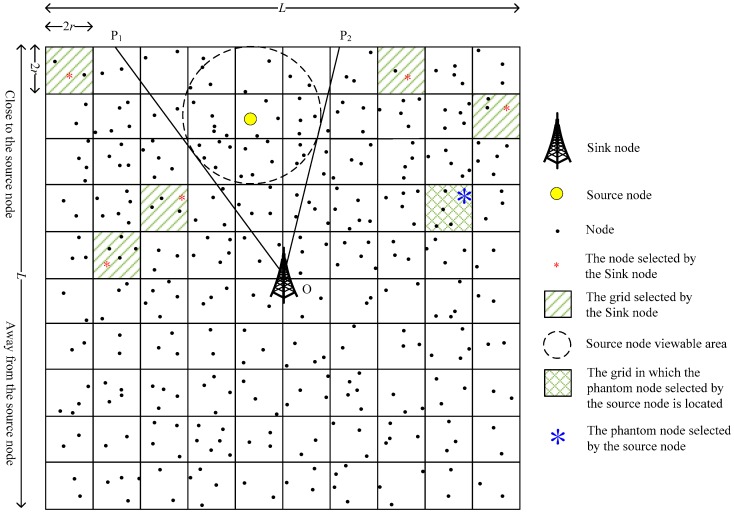
Grid simulation.

**Figure 5:**
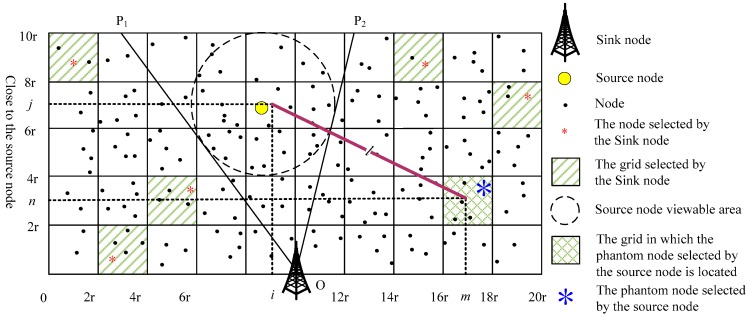
Simplified grid.

Taking the SoN as an example, the specific process is as follows:

(1) The SoN randomly selects a PN from the PNCS and removes the PN ID from the PNCS, which means that a PN can only be used once.

(2) Once selecting the PN, the SoN can simulate the grid diagram, as shown in Figure 4, and determine their positions in the grids according to the grid numbers of itself and the PN. The SoN simplifies Figure 4 to Figure 5 and builds a coordinate system. As shown in Figure 5, the SoN is in grid $G_{i\times j}$, and the PN is in grid $G_{m\times n}$.

(3) According to its grid number $G_{i\times j}$ and the PN’s grid number $G_{m\times n}$, the SoN obtains the coordinates of the center point of the grids are (*i*, *j*) and (*m*, *n*), respectively. Then it calculates two-point straight line *l*, as shown in Equation (4),

(4) $$\frac{x-i}{m-i}=\frac{y-j}{n-j}.$$

(4) The SoN determines whether the slope of the line *l* is 0. If the slope is 0, that is, the grids where the SoN and the PN belong to are in the same row, the SoN searches its neighbor table for the set of neighbor nodes in the direction of the target grid, and randomly selects a node as the next hop node. The subsequent nodes do the same operation until the data packet reaches the PN.

(5) If the slope of the line *l* is not 0, the SoN calculates x=i±1, y=j±1, so that the distance from (*x*, *y*) to line *l* does not exceed $\sqrt{2}$*r. For example, as shown in Figure 5, if there are two possibilities (right grid and bottom right grid), the left and the right grids are determined first. The SoN makes x=i+1, y=j, that is to say, the SoN determines the next grid through which the data packet passes is the right grid. Then it searches its neighbor table to find the neighbor node set in the right grid, and randomly selects one node as the next hop node.

The subsequent nodes repeat steps (4) and (5) until the data packet is transmitted to the PN.

#### 3.3.2. Step 2: The Phantom Node Sends Data Packets to The Sink Node

The PN adopts the single-path routing method [27] to forward data packets to the SN. More specifically, after receiving the data packet, the PN randomly selects one node from the near-hop neighbor nodes as the next hop node, and forwards the packet to it. The next hop node repeats this process until the data packet reaches the SN.

**Figure 6:**
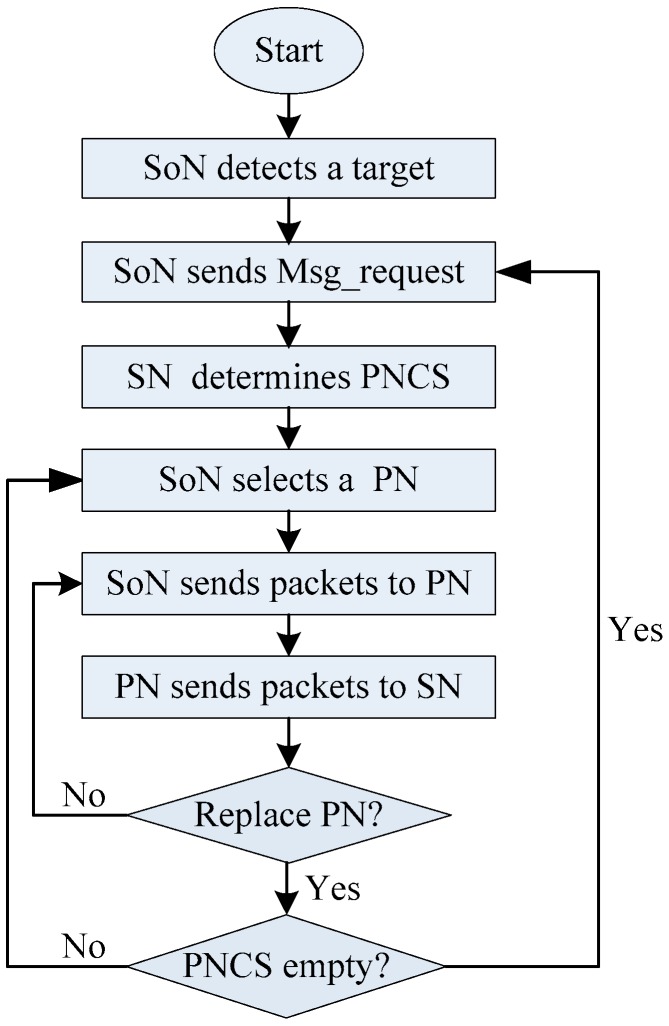
The flowchart of our proposed SPS scheme.

Figure 6 shows the flowchart of our proposed SPS scheme. 

In References [20,21], the PN distribution diversity and phantom routing path randomness are used as important indicators for security performance analysis. In this paper, it is the sink node that helps the source node choose the PNs randomly, and the phantom routing path is determined only after the packet transmission direction is determined through the PN. Moreover, the next hop node in the target grid is also randomly selected, so the PN distribution and the phantom routing path are maximally randomized and diversified.

## 4. DPS: Grid-Based Dual Phantom Node Source Location Privacy Protection Scheme

**Figure 7:**
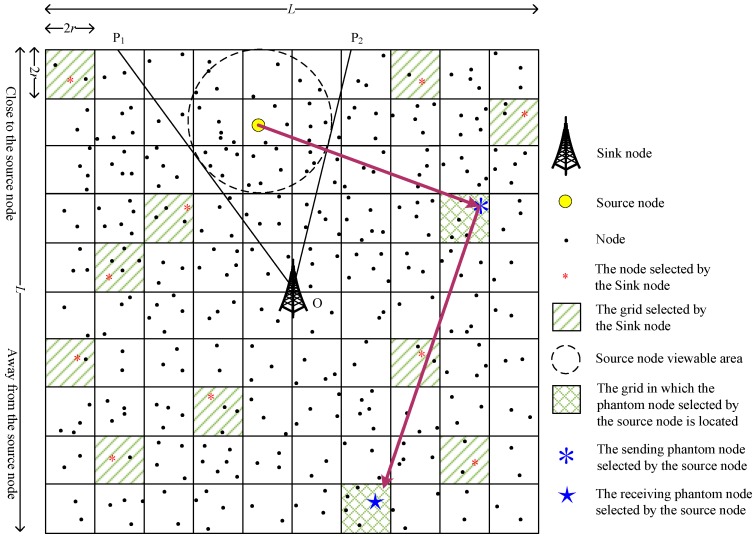
The same side view of the phantom nodes.

**Figure 8:**
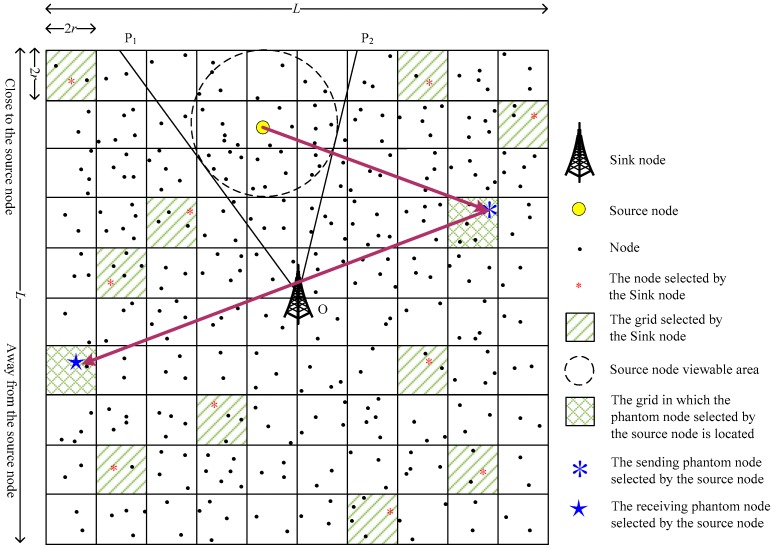
The opposite side view of the phantom nodes.

In order to explore the impact of the number of simultaneously used PNs on the performance of the scheme, we further propose a grid-based dual phantom node source location privacy protection scheme (DPS). Compared with the SPS scheme, DPS uses two types of PN simultaneously: The sending phantom node (SPN) and the receiving phantom node (RPN). The SPN is close to the SoN, and the RPN is far from the SoN, as shown in Figure 7 and Figure 8.

The simulation results show that DPS can provide higher security performance for the network, but its communication overhead also increases. In practical applications, it is necessary to choose a suitable solution according to specific performance requirements.

### 4.1. The Initialization Phase

The network initialization phase of the DPS scheme is the same as that of the SPS scheme. After the initialization is completed, each node establishes its own neighbor table based on the received broadcast message.

### 4.2. The Phantom Node Determination Phase

(1) The first step in the PN determination phase is the same as that of the SPS scheme;

(2) After the SN receives the request packet from the SoN, it randomly selects 2*M* grids from the grids outside the visible region of the SoN, and randomly selects a node from each grid to form two PNCSs: The SPNCS and the RPNCS. The SPNCS is composed of nodes selected from *M* grids close to the SoN, and the RPNCS is composed of nodes selected from *M* grids far from the SoN;

(3) The SN sends the two PNCSs and the grid number of each node in them to the SoN;

(4) Once receiving the PNCSs, the SoN randomly selects a node from the SPNCS and a node from the RPNCS as the SPN and the RPN, respectively.

### 4.3. The Routing Phase

In the routing phase, the SoN sends the data packets to the SN periodically through the selected two PNs. The data packets need to go through three transmission steps: The SoN sends the data packets to the SPN, the SPN sends the data packets to the RPN, and the RPN sends the data packets to the SN.

(1) Step 1: The source node sends data packets to the sending phantom node:

The process that the SoN sends data packets to the SPN is the same as described in Section 3.3.1;

(2) Step 2: The sending phantom node sends data packets to the receiving phantom node:

Since the SPN and the RPN are randomly selected by the SoN, according to whether the SPN and the RPN are on the same side of the SN, there are two possibilities: The same side (as shown in Figure 7, the SPN and RPN are on the same side of the SN) and the opposite side (as shown in Figure 8, the SPN and RPN are on different sides of the SN). When it is the same side case, the transmission path is determined using the operations described in Section 3.3.1. When it is the opposite side case, the data packets may pass through the SN when they are transmitted from the SPN to the RPN, and the DPS scheme will degenerate to the SPS scheme. In order to avoid the above situation and ensure the privacy of the source location, we propose that the SPN first transmits the data packets to a transition node whose row (or column) is the same as that of the RPN, and then the transition node transmits the data packets to the RPN;

(3) Step 3: The receiving phantom node sends data packets to the sink node:

The RPN transmits the data packets to the SN using the single-path routing method described in Section 3.3.2.

**Figure 9:**
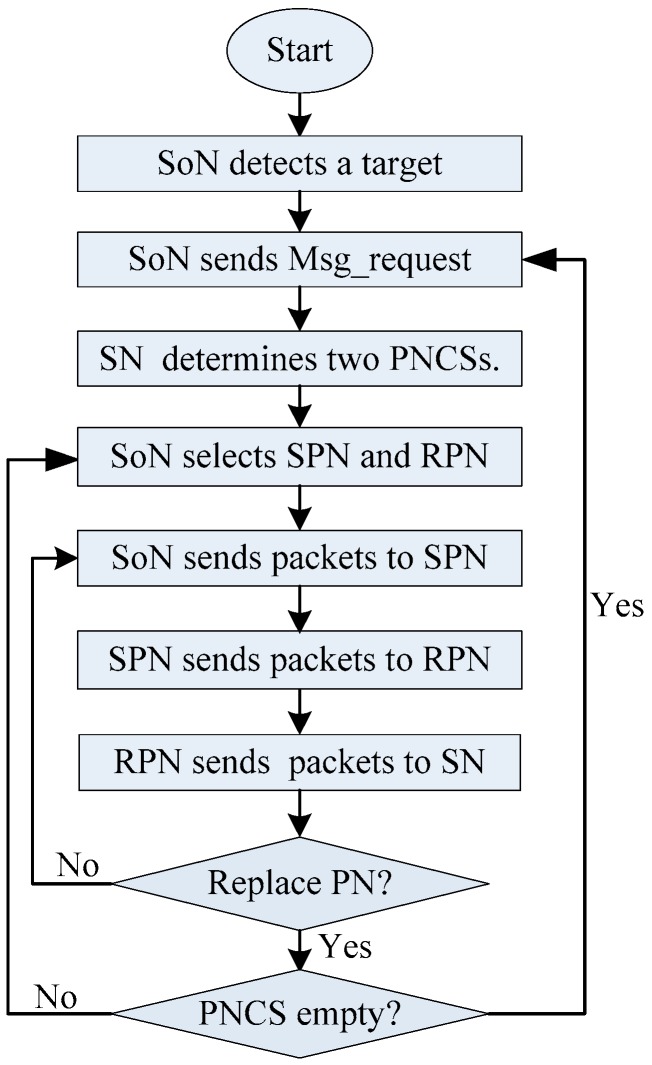
The flowchart of our proposed DPS scheme.

Figure 9 shows the flowchart of our proposed DPS scheme. 

## 5. Performance Analysis and Simulation

We compare our proposed schemes with RPBMP [16], EPUSBRF [18] and the shortest path algorithm from two aspects of security performance and communication overhead. According to References [16,18], EPUSBRF and RPBMP only include the PN determination phase, and they do not consider the lasting time of the PN and the number of PN. Therefore, we analyze the case of single PN based on the descriptions in References [16,18]. In order to prove the performance of our proposed schemes more intuitively, the shortest path algorithm with the lowest energy consumption is selected as one of the comparison schemes. Since the shortest path algorithm is locally optimal in terms of communication overhead, it does not consider security performance, so no further analysis is done below. For the convenience of description, in the following performance analysis, we assume that the source node visible area radius is the same as the node communication radius.

### 5.1. Security Performance Analysis

For an attacker, he needs to trace to the PN so as to find the real source node. More PNs can bring more diversities and uncertainties in routing which will increase the difficulty for the attacker to track back and provide longer safety period for the true source node. In this paper, the security performance indicator is also represented by the safety period. The safety period in our latter simulations is represented by the number of packets sent by the source node before the panda is captured.

#### 5.1.1. Security Performance Analysis of EPUSBRF, RPBMP and Shortest Path Algorithms

Suppose that the time for the attacker to trace back one hop along the routing path is *T*. For the EPUSBRF scheme, let the hop count from the SoN to the PN be ${\mathrm{Hop}}_{s,p}$ and the hop count from the PN to the SN be ${\mathrm{Hop}}_{p,\mathrm{sink}}$. For the RPBMP scheme, let the hop count from the SoN to the PN be ${{\mathrm{Hop}}^{\prime }}_{s,p}$ and the hop count from the PN to the SN be ${{\mathrm{Hop}}^{\prime }}_{p,\mathrm{sink}}$. For the shortest path algorithm, let the hop count from the SoN to the SN be ${\mathrm{Hop}}_{s,\mathrm{sink}}$.

For EPUSBRF and RPBMP, the routing path hops are given by Equations (5) and (6), respectively:

(5) $${\mathrm{Hop}}_{\mathrm{EPUSBRF}}{=\mathrm{Hop}}_{s,p}{+\mathrm{Hop}}_{p,\mathrm{sink}},$$

(6) $${\mathrm{Hop}}_{\mathrm{RPBMP}}={{\mathrm{Hop}}^{\prime }}_{s,p}+{{\mathrm{Hop}}^{\prime }}_{p,\mathrm{sink}},$$

For the shortest path algorithm, the routing path hops is:

(7) $${\mathrm{Hop}}_{\mathrm{Shortest}}{=\mathrm{Hop}}_{s,\mathrm{sink}}.$$

For EPUSBRF and RPBMP, the time required for the attacker to trace back to the SoN is given by Equations (8) and (9), respectively:

(8) $$T_{\mathrm{EPUSBRF}}{=\mathrm{Hop}}_{\mathrm{EPUSBRF}}*{T=(\mathrm{Hop}}_{s,p}+{\mathrm{Hop}}_{p,\mathrm{sink}})*T,$$

(9) $$T_{\mathrm{RPBMP}}={\mathrm{Hop}}_{\mathrm{RPBMP}}{*T=({\mathrm{Hop}}^{\prime }}_{s,p}+{{\mathrm{Hop}}^{\prime }}_{p,\mathrm{sink}})*T.$$

For the shortest path algorithm, the time required for the attacker to trace back to the SoN is shown in Equation (10):

(10) $$T_{\mathrm{Shortest}}{=\mathrm{Hop}}_{\mathrm{Shortest}}{*T=\mathrm{Hop}}_{s,\mathrm{sink}}*T.$$

#### 5.1.2. Security Performance Analysis of our Proposed Schemes

The safety period is the number of packets the SoN has sent before it is caught by the attacker. In our proposed schemes, the safety period is mainly related to the PN usage time $T_{\mathrm{pp}}$, the number of nodes *M* in the PNCS, and the number of routing path hops ${\mathrm{Hop}}_{\mathrm{sum}}$.

(1) Phantom Node Usage Time $T_{\mathrm{pp}}$

In Reference [19], in order to prevent the attacker from tracing back to the SoN, it is proposed that the lasting time of each routing path should be less than the time used by the attacker to trace back to the SoN. In this paper, in order to ensure the location privacy of the source node and use the routing path efficiently, the PN usage time is determined according to the time required for the attacker to trace to the PN.

Suppose that the SoN generates a data packet every time interval *T*. In the attack model, it is assumed that the attacker quickly moves to the sending node once it overhears a packet, that is, the time that the attacker traces back one hop along the routing path is *T*. Therefore, **Conclusion 1** can be drawn.

**Conclusion** **1.** Suppose that in our proposed NRSPS scheme, the hop count from the source node to the phantom node is ${\mathrm{Hop}}_{s,\mathrm{ps}}$, and the hop count from the phantom node to the sink node is ${\mathrm{Hop}}_{\mathrm{ps},\mathrm{sink}}$; in the NRDPS scheme, the hop count from the source node to the PN is the same as in the NRSPS scheme, also is ${\mathrm{Hop}}_{s,\mathrm{ps}}$, the hop count from the SPN to the RPN is ${\mathrm{Hop}}_{\mathrm{ps},\mathrm{pr}}$, the hop count from the RPN to the sink node is ${\mathrm{Hop}}_{\mathrm{pr},\mathrm{sink}}$, and the data packet interval is *T*.

The time required for the attacker to trace to the PN in the NRSPS scheme is:

(11) $$T_{\mathrm{NRSPSp}}{=T*(\mathrm{Hop}}_{\mathrm{ps},\mathrm{sink}}).$$

The time required for the attacker to trace to the SoN in the NRSPS scheme is:

(12) $$T_{\mathrm{NRSPSs}}{=T*(\mathrm{Hop}}_{s,\mathrm{ps}}{+\mathrm{Hop}}_{\mathrm{ps},\mathrm{sink}}).$$

The time required for the attacker to trace to the SPN in the NRDPS scheme is:

(13) $$T_{\mathrm{NRDPSp}}{=T*(\mathrm{Hop}}_{\mathrm{ps},\mathrm{pr}}{+\mathrm{Hop}}_{\mathrm{pr},\mathrm{sink}}).$$

The time required for the attacker to trace to the SoN in the NRDPS scheme is:

(14) $$T_{\mathrm{NRDPSs}}{=T*(\mathrm{Hop}}_{s,\mathrm{ps}}{+\mathrm{Hop}}_{\mathrm{ps},\mathrm{pr}}{+\mathrm{Hop}}_{\mathrm{pr},\mathrm{sink}}).$$

**Conclusion** **2.** Suppose that a phantom node usage time is $T_{\mathrm{pp}}$. $T_{\mathrm{pp}}$ is represented as $T_{\mathrm{NRSPSpp}}$ in the NRSPS scheme and $T_{\mathrm{NRDPSpp}}$ in the NRDPS scheme. The NRSPS scheme and the NRDPS scheme only need to satisfy the Equations (15) and (16) respectively to ensure that the attacker cannot trace back to the phantom node, thus ensuring that the attacker cannot trace back to the source node. In very special cases, even if the attacker traces back to the phantom node, the attacker still needs to backtrack ${\mathrm{Hop}}_{s,\mathrm{ps}}$ to capture the source node.

(15) $$T_{\mathrm{NRSPSpp}}\leq T_{\mathrm{NRSPSp}}{=(\mathrm{Hop}}_{\mathrm{ps},\mathrm{sink}})*T$$

(16) $$T_{\mathrm{NRDPSpp}}\leq T_{\mathrm{NRDPSp}}{=(\mathrm{Hop}}_{\mathrm{ps},\mathrm{pr}}{+\mathrm{Hop}}_{\mathrm{pr},\mathrm{sink}})*T$$

(2) The Number of Nodes *M* in the Phantom Node Candidate Set

In the NRDPS scheme, the sink node randomly selects *M* grids from near source node side and *M* grids from away the source node side, respectively. Then the SN randomly selects one node from each grid to form the sending phantom node candidate set (SPNCS) and the receiving phantom node candidate set (RPNCS). Finally, the SoN randomly selects *i* pair node as the SPN and the RPN.

**Conclusion** **3.** Suppose that the panda’s stay time is $T_{1}$, and the number of nodes in the phantom node candidate set is M, Equations (17) and (18) can be derived according to Equations (15) and (16).

(17) $$T_{l}\leq {M*T}_{\mathrm{NRSPSpp}}\leq {M*T}_{\mathrm{NRSPSp}}=M*\left({\mathrm{Hop}}_{\mathrm{ps},\mathrm{sink}}\right)*T$$

(18) $$T_{l}\leq {M*T}_{\mathrm{NRDPSpp}}\leq {M*T}_{\mathrm{NRDPSp}}=M*\left({\mathrm{Hop}}_{\mathrm{ps},\mathrm{pr}}{+\mathrm{Hop}}_{\mathrm{pr},\mathrm{sink}}\right)*T$$

From Equations (17) and (18), we can see that when $\frac{T_{l}}{T_{\mathrm{pp}}}\leq M$, the attacker cannot capture the SoN, where $T_{\mathrm{pp}}$ in the NRSPS scheme is shown in Equation (15), and $T_{\mathrm{pp}}$ in the NRDPS scheme is shown in Equation (16).

(3) Routing Path Hops ${\mathrm{Hop}}_{\mathrm{sum}}$

According to the assumption of **0**, the hops of routing path ${\mathrm{Hop}}_{\mathrm{sum}}$ of the NRSPS scheme and NRDPS scheme can be derived by Equations (19) and (20), respectively:

(19) $${\mathrm{Hop}}_{\mathrm{NRSPSsum}}{=\mathrm{Hop}}_{s,\mathrm{ps}}{+\mathrm{Hop}}_{\mathrm{ps},\mathrm{sink}}$$

(20) $${\mathrm{Hop}}_{\mathrm{NRDPSsum}}{=\mathrm{Hop}}_{s,\mathrm{ps}}{+\mathrm{Hop}}_{\mathrm{ps},\mathrm{pr}}{+\mathrm{Hop}}_{\mathrm{pr},\mathrm{sink}}$$

Since the attacker can only hop-by-hop backtrack, it takes time $T_{s}{=T*\mathrm{Hop}}_{\mathrm{sum}}$ to capture the SoN. According to Equations (15)–(20), the usage time of the PN $T_{\mathrm{pp}}\leq$ the time for the attacker to trace to the PN $T_{\mathrm{ps}}<$ the time for the attacker to trace to the SoN $T_{s}$. Therefore, before the attacker traces to the PN, the SoN has updated the PN and the attacker cannot continue backtracking.

#### 5.1.3. Comparison of Security Performance

(1) The Case of Not Replacing the Phantom Node

Suppose that the NRSPS scheme, the NRDPS scheme, the EPUSBRF and the RPBMP use the same network topology. The PNs are all represented as p_c_, and the path of the data packets transmitted from p_c_ to the SN does not pass through the visible area of the source node. The time that the attacker traces back one hop along the routing path is T. Since the purpose of using the PN is to hide the source node so that the attacker can only trace back to the PN while he cannot trace back to the real source node. Here, only the security performance is comparatively analyzed.

The time required for the attacker to trace to the PN in the EPUSBRF scheme is:

(21) $$T_{\mathrm{EPUSBRFt}}=\left({\mathrm{Hop}}_{\mathrm{pc},\mathrm{sink}}\right)$$

Suppose that the same-hop routing threshold in RPBMP is $h_{\mathrm{RPBMP}}$, the time required for the attacker to trace to the PN is:

(22) $$T_{\mathrm{RPBMPt}}=\left({\mathrm{Hop}}_{\mathrm{pc},\mathrm{sink}}{+h}_{\mathrm{RPBMP}}\right)$$

The time required for the attacker to trace to the PN in the NRSPS scheme is:

(23) $$T_{\mathrm{NRSPSt}}=\left({\mathrm{Hop}}_{\mathrm{pc},\mathrm{sink}}\right)*T$$

The time required for the attacker to trace to the PN in the NRDPS scheme is:

(24) $$T_{\mathrm{NRDPSt}}=\left({{\mathrm{Hop}}^{\prime }}_{\mathrm{pc},\mathrm{sink}}\right)*T$$

where ${{\mathrm{Hop}}^{\prime }}_{\mathrm{pc},\mathrm{sink}}{=\mathrm{Hop}}_{\mathrm{ps},\mathrm{pr}}{+\mathrm{Hop}}_{\mathrm{pr},\mathrm{sink}}$.

Therefore, in the above assumption, $T_{\mathrm{NRDPSt}}>T_{\mathrm{RPBMPt}}\geq T_{\mathrm{EPUSBRFt}}=T_{\mathrm{NRSPSt}}$, and the equal sign is established when h_RPBMP_ = 0.

(2) The Case of Replacing the Phantom Node

In this paper, *M* PNs are used in total. After the PN is replaced, the data packet transmission path will also change, and the attacker cannot eavesdrop the data packet and will return to the SN along the original path. The time required for the attacker to trace to the last PN in the RSPS scheme is:

(25) $$T_{\mathrm{RSPSt}}=\left({\mathrm{Hop}}_{\mathrm{pc},\mathrm{sink}}\right){*T*M+T}_{\mathrm{Esum}},$$

where $T_{\mathrm{Esum}}$ represents the total time taken by the attacker to return to the SN when he cannot overhear the data packets. The time required for the attacker to trace to the last PN in the RDPS scheme is:

(26) $$T_{\mathrm{RDPSt}}=\left({{\mathrm{Hop}}^{\prime }}_{\mathrm{pc},\mathrm{sink}}\right){*T*M+T}_{\mathrm{Esum}},$$

where ${{\mathrm{Hop}}^{\prime }}_{\mathrm{pc},\mathrm{sink}}={\mathrm{Hop}}_{\mathrm{ps},\mathrm{pr}}+{\mathrm{Hop}}_{\mathrm{pr},\mathrm{sink}}$.

In summary, we can obtain:

(27) $$T_{\mathrm{RDPSt}}>T_{\mathrm{RSPSt}}>T_{\mathrm{NRDPSt}}>T_{\mathrm{RPBMPt}}\geq T_{\mathrm{EPUSBRFt}}=T_{\mathrm{NRSPSst}}\gg T_{\mathrm{Shortest}}.$$

Therefore, the security performance of our proposed RDPS scheme is superior to the RSPS scheme and better than the RPBMP, EPUSBRF and the shortest path algorithms.

### 5.2. Analysis of Communication Overhead

Because the energy of WSNs is limited and lower energy consumption means a higher lifetime of the WSNs, so energy consumption is another key factor in WSNs. In this paper, the energy consumption is represented by communication overhead.

Suppose that the communication overhead for each node to forward a data packet is the same. The communication overhead in this paper is represented by the number of nodes through which the data packets pass. Since the SN determines the PNCS in this paper, the SN is usually wired and has powerful hardware resources, the communication overhead of the SN is usually not considered in related researches. At the same time, there are initialization phases in all of the related researches and the costs are similar. Hence, we take the same approach as Reference [18], and do not analyze the communication overhead of initialization phase. Therefore, we mainly analyze the communication overhead of the phases of determining PN and the routing.

#### 5.2.1. Communication Overhead of the Phantom Node Determination Phase

In the existing source node location privacy protection schemes, the PN is determined by the SoN via flooding, which will consume a lot of communication overheads. We show the reason for the following mathematical analysis.

In the EPUSBRF scheme, the PN is determined by the SoN using *h*-hop flooding and the *h*-hop directed routing. According to the network environment described in Reference [18], the average neighbor numbers of each node is 8.64, so the communication overhead of determining PN in EPUSBRF scheme is ${\mathrm{Hop}}_{\mathrm{pEPUSBRF}}=8.64+8.64^{2}+\cdots +8.64^{h}+h$ where the communication overhead for *h*-hop flooding is $8.64+8.64^{2}+\cdots +8.64^{h}$, and the communication overhead for *h*-hop directed routing is *h*.

According to Reference [16], the communication overhead of determining PN in RPBMP scheme is ${\mathrm{Hop}}_{\mathrm{pRPBMP}}=3*h$. The shortest path algorithm does not need to determine PN, so the communication overhead of determining PN in it is ${\mathrm{Hop}}_{\mathrm{pShortest}}=0$.

However, in our proposed schemes, we use the sink node to help the source node to determine the PN. As the energy of the sink node is unlimited, its communication overhead is not considered in related researches. Therefore, the communication overhead of determining PN in our proposed schemes is ${\mathrm{Hop}}_{\mathrm{pSPS}}={\mathrm{Hop}}_{\mathrm{pDPS}}=0$.

In summary, the following relationships are obtained:

(28) $${0=\mathrm{Hop}}_{\mathrm{pShortest}}={\mathrm{Hop}}_{\mathrm{pSPS}}={\mathrm{Hop}}_{\mathrm{pDPS}}<{\mathrm{Hop}}_{\mathrm{pRPBMP}}<{\mathrm{Hop}}_{\mathrm{pEPUSBRF}}.$$

Therefore, in the phase of determining PN, the communication overhead of our proposed schemes is the same as the shortest path algorithm, and both are lower than that of the RPBMP and EPUSBRF schemes.

#### 5.2.2. Communication Overhead of the Routing Phase

Suppose that the SPS, EPUSBRF and RPBMP schemes use the same network topology, and the PNs are all represented as p_c_, and the path of the data packets transmitted from p_c_ to the SN does not pass through the visible area of the source node.

The communication overhead of the EPUSBRF scheme in the routing phase is

(29) $${\mathrm{Hop}}_{\mathrm{EPUSBRFc}}{=\mathrm{Hop}}_{\mathrm{pc},\mathrm{sink}}.$$

The communication overhead of the RPBMP scheme in the routing phase is

(30) $${\mathrm{Hop}}_{\mathrm{RPBMPc}}{=3*\mathrm{Hop}}_{\mathrm{pc},\mathrm{sink}}.$$

The communication overhead of the NRSPS scheme in the routing phase is

(31) $${\mathrm{Hop}}_{\mathrm{NRSPSc}}={\mathrm{Hop}}_{\mathrm{pc},\mathrm{sink}}.$$

The communication overhead of the RSPS scheme in the routing phase is

(32) $${\mathrm{Hop}}_{\mathrm{RSPSc}}={M*\mathrm{Hop}}_{\mathrm{pc},\mathrm{sink}}.$$

The communication overhead of the NRDPS scheme in the routing phase is

(33) $${\mathrm{Hop}}_{\mathrm{NRDPSc}}=\left({\mathrm{Hop}}_{\mathrm{pc},\mathrm{pr}}+{\mathrm{Hop}}_{\mathrm{pr},\mathrm{sink}}\right).$$

The communication overhead of the RDPS scheme in the routing phase is

(34) $${\mathrm{Hop}}_{\mathrm{RDPSc}}=M*\left({\mathrm{Hop}}_{\mathrm{pc},\mathrm{pr}}+{\mathrm{Hop}}_{\mathrm{pr},\mathrm{sink}}\right).$$

In summary, the following relationships are obtained:

$${\mathrm{Hop}}_{\mathrm{RPBMPc}}>{\mathrm{Hop}}_{\mathrm{RDPSc}}={\mathrm{Hop}}_{\mathrm{NRDPSc}}>{\mathrm{Hop}}_{\mathrm{EPUSBRFc}}{=\mathrm{Hop}}_{\mathrm{RSPSc}}{=\mathrm{Hop}}_{\mathrm{NRSPSc}},\;M=1$$

$${\mathrm{Hop}}_{\mathrm{RPBMPc}}{>\mathrm{Hop}}_{\mathrm{RDPSc}}>{\mathrm{Hop}}_{\mathrm{RSPSc}}>{\mathrm{Hop}}_{\mathrm{NRDPSc}}{>\mathrm{Hop}}_{\mathrm{EPUSBRFc}}{=\mathrm{Hop}}_{\mathrm{NRSPSc}},\;M=2$$

$${\mathrm{Hop}}_{\mathrm{RDPSc}}>{\mathrm{Hop}}_{\mathrm{RSPSc}}\geq {\mathrm{Hop}}_{\mathrm{RPBMPc}}{>\mathrm{Hop}}_{\mathrm{NRDPSc}}{>\mathrm{Hop}}_{\mathrm{EPUSBRFc}}{=\mathrm{Hop}}_{\mathrm{NRSPSc}}.\;M\geq 3$$

Therefore, as the number of PNs actually used increases, the communication overheads of the RSPS scheme and the RDPS scheme increase accordingly.

#### 5.2.3. Comparison of the Total Communication Overhead

The total communication overhead is the sum of the communication overheads consumed in the PN determination phase and the routing phase. The total communication overhead of each scheme is shown in Table 3. It can be seen from Table 3 that when M = 1,

(35) $${\mathrm{Hop}}_{\mathrm{EPUSBRFsum}}>{\mathrm{Hop}}_{\mathrm{RPBMPsum}}>{\mathrm{Hop}}_{\mathrm{RDPSsum}}{=\mathrm{Hop}}_{\mathrm{NRDPSsum}}>{\mathrm{Hop}}_{\mathrm{RSPSsum}}={\mathrm{Hop}}_{\mathrm{NRSPSsum}}$$

Therefore, our proposed RSPS and NRSPS schemes have the lowest total communication overhead among the seven schemes because no communication overhead is generated in the PN determination phase. The total communication overhead of our proposed NRDPS and RDPS schemes is higher than that of the RSPS and NCPSP schemes, due to the actual use of dual PNs. The EPUSBRF scheme has the largest total communication overhead because the communication overhead increases exponentially, due to the flooding in the PN determination phase. Therefore, it can be seen that the total communication overhead of our proposed schemes is lower than that of the EPUSBRF and RPBMP schemes.

### 5.3. Comparison of the Performance Simulation

In order to verify the performance of our proposed schemes, we perform simulations with Matlab platform on the safety period and communication overhead. We compare our proposed schemes with RPBMP [16], EPUSBRF [18] and the shortest path algorithm. In order to facilitate the comparison of the performance of each scheme, we follow the simulation scenario of [18].

Suppose that 10,000 nodes are randomly and evenly distributed in the area of 6000 m*6000 m, and the communication radius of each node is 110 m. The entire network is divided into 3600 grids, each with a unit length of 100 m. The SN is fixed at the center of the network, and the SoN is randomly selected from the nodes in the network which corresponds to the scenario that the panda pops up at a random location. The attacker’s hearing radius is the same as the sensor node’s communication radius, and the visible area radius is set to be 110 m. In the *h*-hop directed routing phase of EPUSBRF and RPBMP, *h* is set to be 10 hops, and the threshold of RPBMP in the same hop routing phase is also *h*.

#### 5.3.1. Comparison of Safety Period

In each experiment, the network topology remains the same, and the SoN sends data packets to the SN at time interval T. The attacker traces back one hop every time he overhears a packet, and the simulation ends once the attacker captures the SoN. If the attacker does not overhead the data packet within the time interval T, he will roll back hop-by-hop along the tracking path until he returns to the SN. If the attacker overhears a packet while returning to the SN, he moves to the packet sending node and continues listening and backtracking until the end of the experiment. It should be noted that the end condition of the experiment in this paper is that the attacker captures the SoN, that is, in a single experiment the panda does not disappear once it appears. If the safety period is greater than 1000, the scheme can ensure that the panda will not be captured by the attacker.

**Experiment 1:** Comparison of the safety period of NRSPS and other three schemes

**Figure 10:**
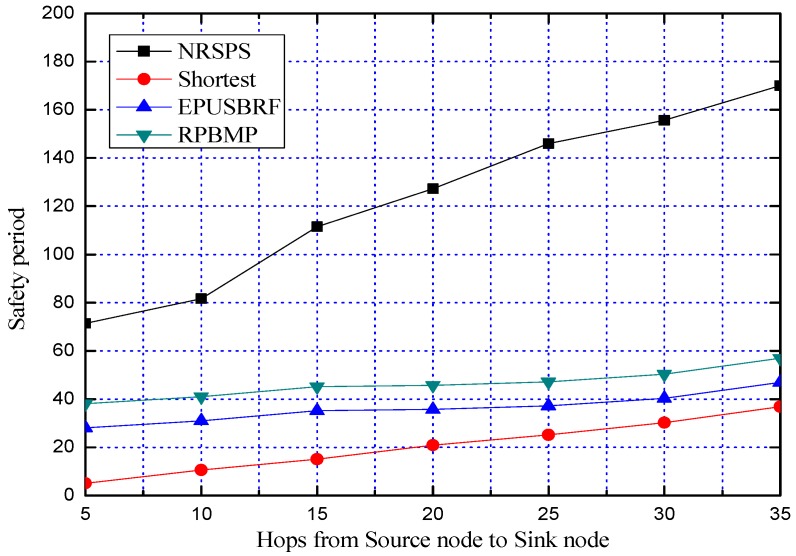
Comparison of the safety period of the NRSPS scheme with the other three schemes.

According to the analysis in Section 5.1.3, the NRSPS scheme provides the shortest safety period among our proposed schemes. Therefore, in Experiment 1, we compare the safety period of the NRSPS scheme with that of the shortest path algorithm, EPUSBRF and RPBMP. The average safety period is obtained by repeating 100 experiments, as shown in Figure 10.

It can be seen from Figure 10 that compared with the shortest path algorithm, EPUSBRF, and RPBMP, the average safety period of our proposed NRSPS scheme increases by 6.08 times, 3.57 times and 2.78 times, respectively. As the hop count between the SoN and the SN increases, the safety period of the four schemes increases. This is because the hop count that the attacker needs to backtrack increases correspondingly with the increase of hop count between the SoN and the SN. However, the safety period of each scheme in the experiment is less than 200, indicating that the SoN only sent less than 200 packets before being captured by the attacker. Therefore, although the safety period of NRSPS scheme improves compared with the shortest path algorithm, EPUSBRF, and RPBMP, it still cannot prevent the SoN from being captured by the attacker.

**Experiment 2:** The effect of the number of phantom nodes on the safety period

**Figure 11:**
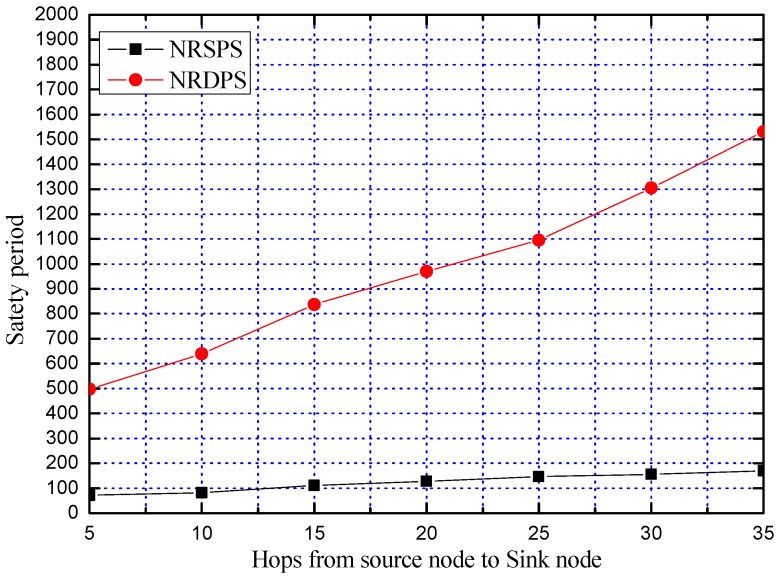
Comparison of the safety period of NRSPS and NRDPS.

Since the NRSPS scheme cannot prevent the source node from being captured by the attacker, in Experiment 2, we consider increasing the number of PNs, and compare the safety period of the NRSPS scheme with that of the NRDPS scheme. The average safety period is obtained by repeating the experiment 100 times, as shown in Figure 11.

It can be seen from Figure 11 that the safety period provided by our proposed NRDPS scheme is much larger than that of the NRSPS scheme. Compared with NRSPS, the average safety period of NRDPS increases by 7.62 times. This is because as the number of PNs increases, the hop count of the routing path increases accordingly, which leads to an increase in the hop count required by the attacker to backtrack. Moreover, when the hop count from the SoN to the SN is greater than 20, the average safety period of the NRDPS scheme is greater than 1000, that is, the attacker is never able to capture the source node.

**Experiment 3:** Comparison of the safety period of the proposed four schemes

**Figure 12:**
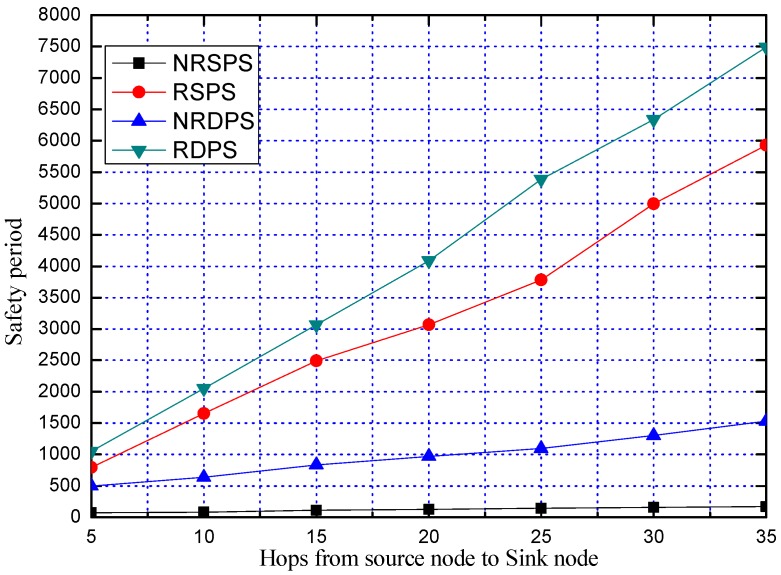
Comparison of the safety period of NRSPS, RSPS, NRDPS and RDPS.

In order to further compare the security performance of our proposed four schemes, we compare the safety period of the four schemes of NRSPS, NRDPS, RSPS and RDPS. The average safety period is obtained by repeating the experiment 100 times, as shown in Figure 12.

It can be seen from Figure 12 that the RDPS scheme has the highest safety period. When the hop count of the source node to the sink node is 5, the safety period of the RDPS scheme reaches 1000, that is, the attacker cannot trace back to the SoN. In practical applications, the SoN is far away from the SN, so the RDPS can ensure the privacy and security of the location of the source node in practical applications. Compared with NRSPS, RSPS and NRDPS schemes, the safety period of the RDPS scheme increases by 32.13 times, 1.33 times and 4.22 times, respectively. In Figure 12, the safety period of the NRSPS does not change much. This is because the safety period of the NRSPS is low, and the change is too small compared with other schemes. Its actual change trend is shown in Figure 10.

It can be seen from the above comparison results that replacing the PN can greatly improve the security performance of the scheme. This is because after the PN is replaced, the attacker loses the interception target. During the process of the attacker returning to the SN, the SoN continues sending the collected data packets to the SN.

**Experiment 4:** The impact of when to replace the phantom node on the safety period

It can be seen from Experiment 3 that replacing the PN can improve the security performance of the scheme. Therefore, in Experiment 4, we further explore the influence of the timing of replacing the PN on security performance. Suppose that in the RDPS scheme, when the attacker backtracks ${\mathrm{Hop}}_{\mathrm{ps},\mathrm{pr}}{+\mathrm{Hop}}_{\mathrm{pr},\mathrm{sink}}/n$ hops, the SoN changes the SPN and the RPN. It can be known from Equations (12) and (13) that in the RDPS scheme, the attacker needs to backtrack ${\mathrm{Hop}}_{\mathrm{sum}}{=\mathrm{Hop}}_{s,\mathrm{ps}}{+\mathrm{Hop}}_{\mathrm{ps},\mathrm{pr}}{+\mathrm{Hop}}_{\mathrm{pr},\mathrm{sink}}$ hops to capture the SoN. In the RDPS scheme, ${\mathrm{Hop}}_{s,\mathrm{ps}}$ is used as the extra security hop count. Suppose that ${\mathrm{Hop}}_{E}$ is the hop count that the attacker backtracks, and when ${\mathrm{Hop}}_{E}{=\mathrm{Hop}}_{\mathrm{ps},\mathrm{pr}}{+\mathrm{Hop}}_{\mathrm{pr},\mathrm{sink}}/n$, the SoN replaces the PN. If the attacker does not overhear the packet, he needs to jump back ${\mathrm{Hop}}_{E}$ hops to the SN. Except for Experiment 4, in other experiments, the value of *n* is 1.

**Figure 13:**
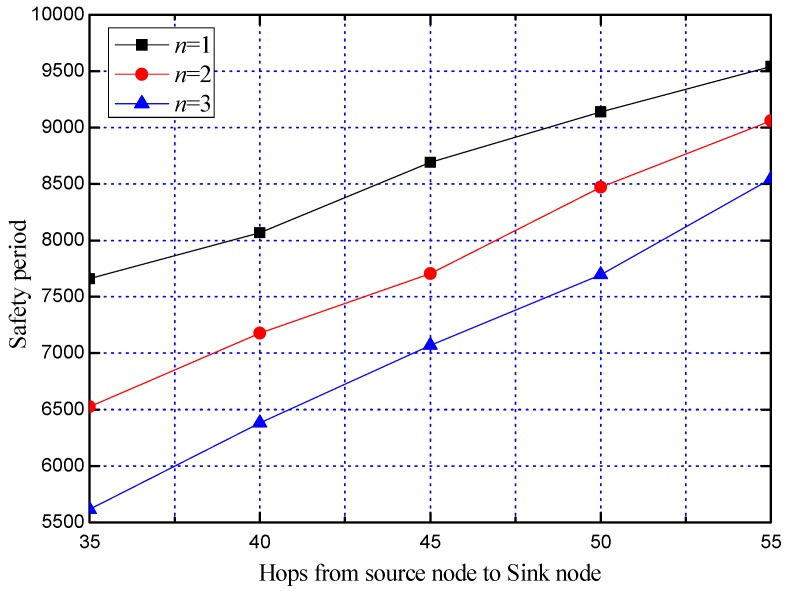
Comparison of the safety period of RDPS scheme with different *n*.

In the case of different *n*, the average safety period is obtained by repeating the experiment 100 times, as shown in Figure 13.

From Figure 13, we can see that the average safety period of the three cases is increased. This is because as the distance between the SoN and the SN increases, the number of hops that the attacker needs to trace back increases accordingly. Meanwhile, it can be seen from Figure 13 that the larger the value of *n* is, the smaller the average safety period is. This is because in the simulation, in order to avoid the experiment falling into an infinite loop, the SoN no longer sends the request packet to the SN even when the PNCS is empty, and the experiment ends when the attacker traces back to the SoN. Therefore, when the number of PNs that can be used is fixed, prematurely changing the PNs will in advance lead to an insufficient of PNs that can be used. As described in Section 3.3.2, if the SoN continues requesting the SN to help determine the PNCS when there is no PN available in the PNCS, better security performance will be obtained.

**Experiment 5:** The impact of *M* on the safety period of the proposed schemes

**Figure 14:**
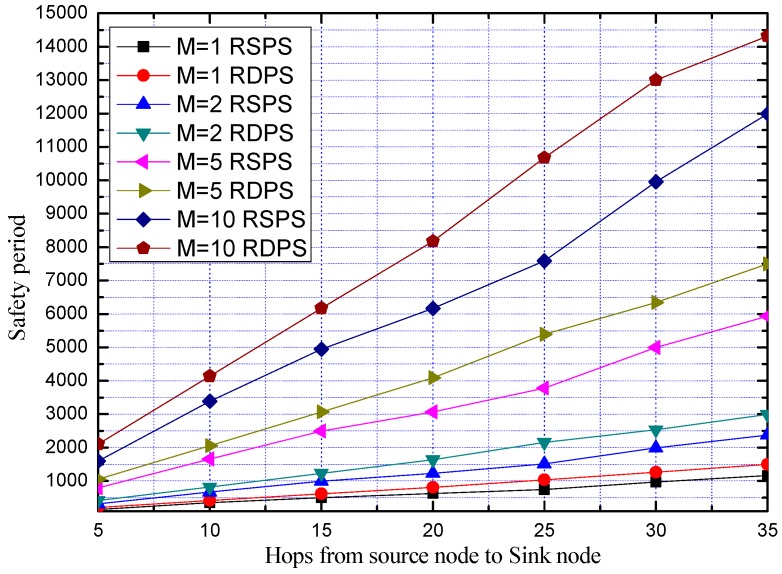
Comparison of the safety period of RSPS and RDPS with different *M*.

In order to further study the impact of *M* on the safety period of the proposed schemes in the case of replacing the PN, we compare the safety period of the two schemes of RSPS and RDPS. The average safety period is obtained by repeating the experiment 100 times, as shown in Figure 14**.**

From Figure 14, we can see that the security performance of our proposed RDPS scheme is superior to the RSPS scheme, and the average safety period of the two schemes increases with *M*. This is because for an attacker, he needs to trace to the PN so as to find the SoN. In fact, the value of M determines the size of the PNCS. The larger the value of M, the more PNs are available for the SoN, and the higher the security is. On the other hand, no matter how big the value of *M* is, the SoN only select one PN from the PNCS for use at a time, after a PN is used for a while, the SoN can replace the PN with another one. The SoN does not use *M* PNs simultaneously. Moreover, after the PN is replaced, the data packet transmission path will also change, and the attacker can no longer overhear the data packets and will roll back to the SN along the previous path. More PNs can bring more diversities and uncertainties in routing which will increase the difficulty for the attacker to track back and provide longer safety period for the true source node.

#### 5.3.2. Comparison of Communication Overhead

**Experiment 6:** Comparison of communication overhead between NRSPS and other three schemes

**Figure 15:**
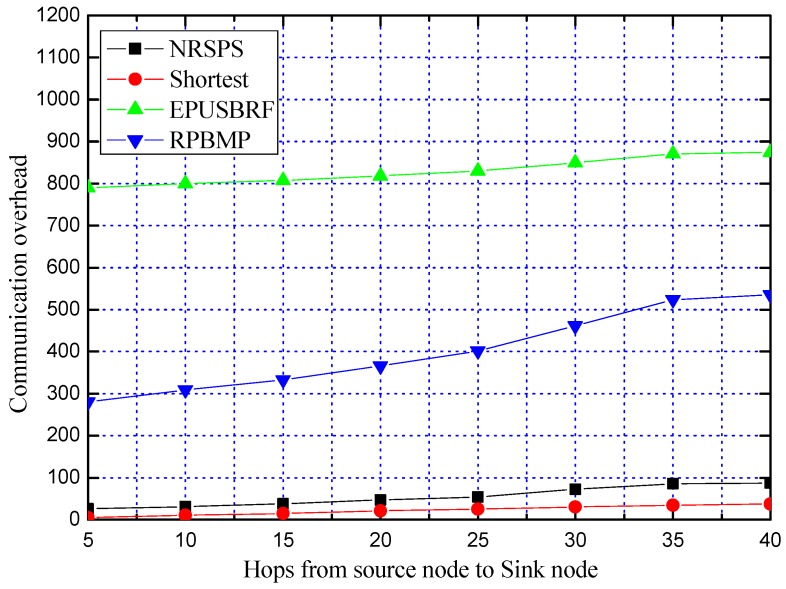
Comparison of communication overheads between NRSPS scheme and other three schemes.

In Experiment 6, we compare the communication overheads of our proposed NRSPS scheme with the shortest path algorithm, EPUSBRF and RPBMP. Since each node consumes the same communication overhead when forwarding packets, the number of nodes through which each packet passes is used as the communication overhead of the routing phase. The total communication overhead is the sum of the communication overheads consumed in the PN phase determination phase and the routing phase. We perform Experiment 6 repeatedly for 100 times and compare its communication overhead with the other three schemes. The simulation results are shown in Figure 15.

It can be seen from Figure 15 that the total communication overhead of our proposed NRSPS scheme is only slightly higher than that of the shortest path algorithm. While compared with EPUSBRF and RPBMP, the total communication overhead of our proposed NRSPS is reduced by 95% and 88%, respectively. The higher communication overhead of EPUSBRF is due to the high communication overhead in the phase of determining PN. In the experiment, *h* is set to be 3 in the flooding phase of EPUSBRF, so the communication overhead for determining PN is 8.64 + 8.642 + 8.643 + 3 = 731.26 (see Section 5.2.1 for theoretical analysis). It can be seen that in the phase of determining PN, the SoN performs flooding to determine the PN which causes a large amount of communication overhead. While in this paper, the SN helps the SoN determine the PN, which saves a lot of communication overhead.

**Experiment 7:** Comparison of communication overhead of our proposed four schemes

**Figure 16:**
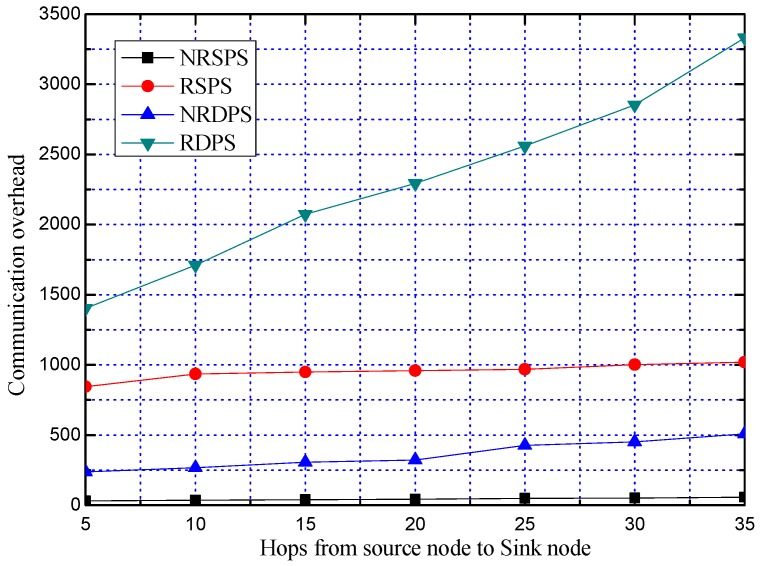
Comparison of the communication overhead of NRSPS, RSPS, NRDPS and RDPS.

We further compare the communication overheads of our proposed four schemes of NRSPS, NRDPS, RSPS and RDPS. The average communication overhead is obtained by repeating the experiment 100 times, as shown in Figure 16.

As can be seen from Figure 16, the communication overhead of the RDPS scheme is the largest, and compared with the NRSPS, RSPS and NRDPS, the communication overhead is increased by 53.1 times, 2.39 times, and 7.10 times, respectively. In Figure 16, the communication overhead of NRSPS does not change much. This is because the communication overhead of NRSPS is low, and the change is too small compared with other schemes. The actual change trend of NRSPS is shown in Figure 16. It can also be seen from Figure 16 that the communication overhead of RSPS and RDPS is greatly increased compared with that of NRSPS and NRDPS. This is because the number of nodes through which the packet passes after the PN is changed increases, resulting in an increase in communication overhead. Although changing the PN can improve the security performance of the scheme, it also causes large communication overhead.

In summary, the communication overhead of the PN determination phase (Overhead 1 for short), the communication overhead of the routing phase (Overhead 2 for short), the total communication overhead (Total overhead for short), and the total safety period are shown in Table 4.

In order to more clearly compare the cost performance of each scheme, we refer to the Safety Period/Communication Cost as the SC ratio. The higher the SC ratio is, the better the performance of the scheme is. It can be seen from Table 4 that the performance of RSPS is better than other schemes. Although the other three schemes proposed in this paper have lower communication overhead or higher safety period, the performance is not balanced. The average total safety period of NRSPS and NRDPS is less than 1000, so the SoN may be captured by the attacker. However, compared with the shortest path algorithm, RPBMP and EPUSBRF, the safety periods of our proposed NRSPS and NRDPS are still greatly improved. The average total safety period of the RDPS is as high as 4087, but the average total communication overhead is as high as 2292.6, which means that it causes a lot of communication overhead while achieving high safety period. Therefore, we can choose the most suitable solution according to different requirements of different scenarios. For example, whether to use single PN or dual PNs, or whether to change the PN.

**Experiment 8:** The impact of *M* on the communication overhead of the proposed schemes

**Figure 17:**
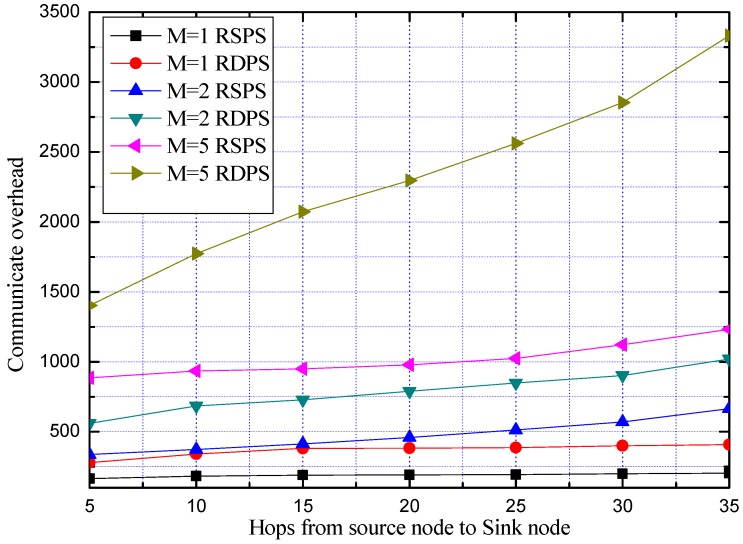
Comparison of communication overhead of RSPS and RDPS with different *M*.

In order to further study the impact of *M* on the communication overheads of the proposed schemes in the case of replacing the PN, we compare the communication overheads of RSPS and RDPS. The average communication overhead is obtained by repeating the experiment 100 times, as shown in Figure 17.

From Figure 17, we can see that the communication overhead of our proposed RDPS is higher than that of the RSPS, due to the actual use of dual PNs, and as *M* increases, the communication overheads of the RSPS scheme and the RDPS scheme increase accordingly.

Although Figure 14 shows that the average safety period of the two schemes increases with *M*, the communication overheads are also increased greatly. Considering the balance between security and network performance, it is necessary to select the most suitable solution according to different requirements of different scenarios.

## 6. Conclusions

Source node location privacy protection is an important issue in widely-used WSNs. In this paper, we propose two new grid-based source location privacy protection schemes in WSNs to ensure the location privacy of source nodes. The sink node with high power resource is used to determine the PNCS, which reduce the total communication overhead. The phantom nodes selected in our proposed schemes can be distributed anywhere in the network and thus have stronger positional randomness. As our proposed schemes ensure the diversification of the routing path by increasing the number of phantom nodes used, the security performance of the source location privacy protection is further improved. The simulation results show that compared with other schemes, our proposed schemes have higher safety period and less communication overhead, thus their application prospect can be expected. Our proposed schemes are especially suitable for resource-constrained scenarios. However, considering the balance of performance of the schemes, it is necessary to select the most suitable solution according to different requirements of different scenarios. For example, when the security requirement for the network is very high and the communication overhead is not a constraint, RDPS can be used. Instead, if the network’s communication conditions are limited and the security requirement is not so high, NRSPS and NRDPS can be adopted.

In the future, we will continue to improve the performance of our schemes, and aim to adapt them to their practical environments. Further research should consider the following aspects:

(1) In this paper, in order to simplify the problem, referring to other relevant pieces in the literature, we evaluate our schemes in the deterministic network model. However, in some practical applications as described in Reference [28], the stochastic deployments should be paid attention too. Therefore, we will study the source node location privacy protection in the stochastic deployments in our subsequent research;

(2) In the network model of this paper, only one source node and one sink node are considered, but in practical applications, there may be multiple source nodes and multiple sink nodes. Hence, in future research, we will investigate the multiple objects tracking scenarios.

## Figures and Tables

**Table 1:** Notations.

Notations	Description
*U*	Node *u*
Hop*_u_*_,sink_	Minimum hop count between node *u* and sink node
L*L	Network size
*R*	Node communication radius
*i*, *j*, *m*, *n*	Grid number index variable
$G_{i\times j}$	Grid number of the *i*-th row and the *j*-th column

**Table 2:** Neighbor table of node *v*.

Node ID	Minimum Hop Count from Sink Node	Grid Number
*u*	${\mathrm{Hop}}_{u,\mathrm{sink}}$	$G_{i\times j}$
…	…	…

**Table 3:** Comparison of the total communication overhead.

Scheme	Total Communication Overhead
EPUSBRF	${\mathrm{Hop}}_{\mathrm{EPUSBRFsum}}{=\mathrm{Hop}}_{\mathrm{pEPUSBRF}}{+\mathrm{Hop}}_{\mathrm{EPUSBRFc}}$
RPBMP	${\mathrm{Hop}}_{\mathrm{RPBMPsum}}{=\mathrm{Hop}}_{\mathrm{pRPBMP}}{+\mathrm{Hop}}_{\mathrm{RPBMPc}}$
Shortest path algorithm	${\mathrm{Hop}}_{\mathrm{Shortest}}{=\mathrm{Hop}}_{\mathrm{pShortest}}{+\mathrm{Hop}}_{s,\mathrm{sink}}$
NRSPS	${\mathrm{Hop}}_{\mathrm{NRSPSsum}}{=\mathrm{Hop}}_{\mathrm{pSPS}}{+\mathrm{Hop}}_{\mathrm{NRSPSc}}$
NRDPS	${\mathrm{Hop}}_{\mathrm{NRDPSsum}}{=\mathrm{Hop}}_{\mathrm{pDPS}}{+\mathrm{Hop}}_{\mathrm{NRDPSc}}$
RSPS	${\mathrm{Hop}}_{\mathrm{RSPSsum}}{=\mathrm{Hop}}_{\mathrm{pSPS}}{+\mathrm{Hop}}_{\mathrm{RSPSc}}$
RDPS	${\mathrm{Hop}}_{\mathrm{DPSsum}}{=\mathrm{Hop}}_{\mathrm{pDPS}}{+\mathrm{Hop}}_{\mathrm{RDPSc}}$

**Table 4:** Comparison of the performance.

		Overhead 1	Overhead 2	Total Overhead	Total Safety Period	SC Ratio
**Shortest path**		0	20.93	20.93	20.93	1
RPBMP		30	335.76	365.76	45.68	0.12
EPUSBRF		731.26	87.05	818.31	35.68	0.04
Proposed	NRSPS	0	43.18	43.18	127.21	0.34
	RSPS	0	959.62	959.62	3070	3.20
	NRDPS	0	323.04	323.04	969	3.00
	RDPS	0	2292.60	2292.60	4087	1.78

## Abbreviations

The following abbreviations are used in this manuscript:

SPS	grid-based Single Phantom node source location privacy protection Scheme
DPS	grid-based Dual Phantom node source location privacy protection Scheme
WSNs	Wireless Sensor Networks
SN	Sink Node
PN	Phantom Node
PNCS	Phantom Node Candidate Set
IoT	Internet of Things
SoN	Source Node
PRS	Phantom Routing Scheme
PSRS	Phantom Single-path Routing Scheme
EPUSBRF	Enhanced source location privacy preservation Protocol Using Source-Based Restricted Flooding
RPBMP	source location privacy preservation Routing Protocol Based on Multi-Path
PRABNS	Phantom Routing based on Area and Brother Neighbor Selecting
RSPS	SPS scheme in the case of Replacing the phantom node
RDPS	DPS scheme in the case of Replacing the phantom node
NRSPS	SPS scheme in the case of Not Replacing the phantom node
NRDPS	DPS scheme in the case of Not Replacing the phantom node
SPN	Sending Phantom Node
RPN	Receiving Phantom Node
SPNCS	Sending Phantom Node Candidate Set
RPNCS	Receiving Phantom Node Candidate Set
Hop_s,p_	Hop count from the Source node to the Phantom node in the EPUSBRF scheme
Hop_p,sink_	Hop count from the Phantom node to the Sink node in the EPUSBRF scheme
Hop’_s,p_	Hop count from the Source node to the Phantom node in the RPBMP scheme
Hop’_p,sink_	Hop count from the Phantom node to the Sink node in the RPBMP scheme
Hop_s,sink_	Hop count from the Source node to the Sink node in the shortest path algorithm
Hop_EPUSBRF_	routing path Hop in the EPUSBRF scheme
Hop_RPBMP_	routing path Hop in the RPBMP scheme
Hop_Shortest_	routing path Hop in the Shortest path algorithm
T_EPUSBRF_	Time required for the attacker to trace back to the source node in the EPUSBRF scheme
T_RPBMP_	Time required for the attacker to trace back to the source node in the RPBMP scheme
T_Shortest_	Time required for the attacker to trace back to the source node in the Shortest path algorithm
T_pp_	Phantom node usage Time
Hop_s,ps_	Hop count from the Source node to the Phantom node in the NRSPS and NRDPS scheme
Hop_ps,sink_	Hop count from the Phantom node to the Sink node in the NRSPS scheme
Hop_ps,pr_	Hop count from the Sending Phantom node to the Receiving Phantom node in the NRDPS scheme
Hop_pr,sink_	Hop count from the Receiving Phantom node to the Sink node in the NRDPS scheme
T_NRSPSp_	Time required for the attacker to trace to the Phantom node in the NRSPS scheme
T_NRSPSs_	Time required for the attacker to trace to the Source node in the NRSPS scheme
T_NRDPSp_	Time required for the attacker to trace to the Phantom node in the NRDPS scheme
T_NRDPSs_	Time required for the attacker to trace to the Source node in the NRDPS scheme
T_NRSPSpp_	Phantom node usage Time in the NRSPS scheme
T_NRDPSpp_	Phantom node usage Time in the NRDPS scheme
Hop_NRSPSsum_	Hops of routing path in the NRSPS scheme
Hop_NRDPSsum_	Hops of routing path in the NRDPS scheme
Hop_pc,sink_	Hop count from the Phantom node to the Sink node in the NRSPS, NRDPS, EPUSBRF and RPBMP scheme
T_EPUSBRFt_	Time required for the attacker to trace to the phantom node in the EPUSBRF scheme
T_RPBMPt_	Time required for the attacker to trace to the phantom node in the RPBMP scheme
T_NRSPSt_	Time required for the attacker to trace to the phantom node in the NRSPS scheme
T_NRDPSt_	Time required for the attacker to trace to the phantom node in the NRDPS scheme
T_RSPSt_	Time required for the attacker to trace to the last phantom node in the RSPS scheme
T_RDPSt_	Time required for the attacker to trace to the last phantom node in the RDPS scheme
Hop_pEPUSBRF_	communication overhead of determining Phantom node in the EPUSBRF scheme
Hop_pRPBMP_	communication overhead of determining Phantom node in the RPBMP scheme
Hop_pShortest_	communication overhead of determining Phantom node in the Shortest path algorithm
Hop_pSPS_	communication overhead of determining Phantom node in the SPS scheme
Hop_pDPS_	communication overhead of determining Phantom node in the DPS scheme
Hop_EPUSBRFc_	Communication overhead of the EPUSBRF scheme in the routing phase
Hop_RPBMPc_	Communication overhead of the RPBMP scheme in the routing phase
Hop_NRSPSc_	Communication overhead of the NRSPS scheme in the routing phase
Hop_RSPSc_	Communication overhead of the RSPS scheme in the routing phase
Hop_NRDPSc_	Communication overhead of the NRDPS scheme in the routing phase
Hop_RDPSc_	Communication overhead of the RDPS scheme in the routing phase
Hop_EPUSBRFsum_	Sum of the communication overheads in the EPUSBRF scheme
Hop_RPBMPsum_	Sum of the communication overheads in the RPBMP scheme
Hop_RDPSsum_	Sum of the communication overheads in the RDPS scheme
Hop_NRDPSsum_	Sum of the communication overheads in the NRDPS scheme
Hop_RSPSsum_	Sum of the communication overheads in the RSPS scheme
Hop_NRSPSsum_	Sum of the communication overheads in the NRSPS scheme
